# Effect of Low Temperature and Nitrogen Modified Atmosphere Treatments on the Storage of High Moisture Indica Rice: Quality, Microstructure, and Metabolome Characteristics

**DOI:** 10.3390/foods14071262

**Published:** 2025-04-03

**Authors:** Yanan Zhao, Lulu Li, Yanfei Li, Yan Zhao

**Affiliations:** School of Food and Strategic Reserves, Henan University of Technology, Zhengzhou 450000, China; 2022920080@stu.haut.edu.cn (Y.Z.); lilulu1023@126.com (L.L.); liyanfei@haut.edu.cn (Y.L.)

**Keywords:** high moisture content rice quality, low temperatures, nitrogen modified atmosphere, untargeted metabolomics

## Abstract

Effects of low temperature (LT, 20 °C) combined with nitrogen-modified atmosphere (MA, 95% N_2_) storage (LT + MA) on quality, microstructure, and metabolome characteristics of high moisture content (15.5%) during 180 days of storage were investigated to explore a potential preservation technique for high moisture rice. The results showed that after 180 days of storage, the fatty acid value, malondialdehyde content, and amylose content of rice under LT + MA storage were 53.33%, 72.93%, and 91.85% of those under conventional storage (CS, conventional atmosphere, 30 °C, RH 65%), respectively. The color, pasting properties, and scanning electron microscopy (SEM) of the LT + MA treatment were found to be markedly superior to those of the CS treatment. In addition, the differential metabolites sucrose, trehalose-6P, trehalose, 3,7-Di-O-methylquercetin (DMQ), rutin, and vitexin 2″-O-rhamnoside (VOR) were screened to assess for sensitivity to changes in storage conditions. The study demonstrated that the LT + MA effectively suppressed the escalation of FAV, MDA content, and amylose content. In addition, it was observed to inhibit the deterioration of color and pasting properties while concurrently maintaining the polygonal shape of rice starch granules. Furthermore, the differential metabolites of non-targeted metabolomics indicated that the LT + MA group exhibited superior efficacy in retarding rice aging.

## 1. Introduction

Rice (*Oryza sativa* L.) is one of the world’s most important staple crops. The world’s primary rice producers are Asian countries such as China and India. Following the harvesting of rice, a proportion of the crop is utilized for processing or direct supply to consumers during the current year. In February 2025, the Food and Agriculture Organization of the United Nations (FAO) published a projection indicating that global rice consumption would reach 537.2 million tons in 2024/25, representing an increase of 1.9% compared to the 2023/24 consumption figures. The remaining portion of rice is stored and rotated to ensure a continuous supply of the commodity. The processing of rice with a high moisture content reduces the production of broken rice, improves the overall yield, and ensures the quality and taste of the final product [1]. When rice with a high moisture content is stored under elevated temperature and oxygen, its fat is very susceptible to hydrolysis and oxidation, resulting in a deterioration of its quality [2]. Subsequent decomposition of the rice fat results in an increase in the content of free fatty acids, thereby altering the composition of fatty acids. This, in turn, has a detrimental effect on the edibility and nutritional quality of the rice. Consequently, it is essential to store high moisture rice properly.

Low temperatures (LT) affect the chemical composition, quality, cooking, and thermal properties of rice, thus slowing the rate of rice aging [3]. LT slows down quality deterioration and nutrient loss by maintaining rice seed vigor and germination, inhibiting the weakening of vitality and the gradual loosening of microstructure, and slowing down the decline in enzyme activity [4]. During the process of rice storage, respiration leads to the depletion of nutrients, including proteins, lipids, and carbohydrates, thereby reducing the nutritional value of the rice. Furthermore, high intensity respiration accelerates the process of oxidation and the internal deterioration of the rice, resulting in lipid and protein peroxidation [5]. Lipid oxidation experienced an acceleration in the presence of elevated temperatures, which consequently gave rise to the generation of peroxides [6]. In addition, low temperatures have been shown to maintain superior quality in rice by effectively curtailing the proliferation of storage pests and microorganisms. Low temperatures have been demonstrated to inhibit the growth of molds and reduce the amount of mycotoxins in grains. For instance, aflatoxin, a mycotoxin, has been shown to not only compromise the quality of grains but also pose a grave threat to human health due to its chronic toxicity, carcinogenicity, mutagenicity, and immunotoxicity [7].

The objective of a modified atmosphere (MA) is to create a low-oxygen concentration that will impact metabolic processes, thereby reducing respiration rates, oxidative stress, and tissue senescence [8]. The primary purpose of storing grain in a modified atmosphere is to kill insects, which can effectively reduce the pollution caused by fumigating with chemical substances [9]. In addition, alternative non-chemical techniques for controlling food pests include using irradiation, dielectric heating, cold plasma treatment, electron beam technology, and soft electrons (low-energy electrons) [9]. Compared to other methods, MA treatment is less expensive, can be applied on a large scale, and is the primary pest treatment method for large grain depots in China. When the nitrogen concentration is 95%, it is effective in preventing insect pests [10].

Low temperature and nitrogen treatments are employed extensively in storing grains, fruits, vegetables, meats, and other agricultural products. The study conducted by Martín et al. [11] demonstrated that the autoxidation of hazelnuts was reduced and the rate of lipid rancidity was slowed down following low temperature and nitrogen storage. The protective effects of low temperature and nitrogen on rice quality may be related to internal metabolic changes under conditions of hypoxia and high temperature stress, which can be investigated by studying metabolomics.

The protective effects of low temperature and nitrogen on rice quality are mainly related to internal metabolic changes under hypoxia and high temperature stress, which can be investigated by studying metabolomics.

The present study was conducted to examine the alterations in FAV, malondialdehyde (MDA) content, amylose content, color, and pasting properties of rice with high moisture content during storage. Furthermore, scanning electron microscopy (SEM) was utilized to examine the microstructure alterations in rice during storage. Subsequently, samples of rice with high moisture content were examined using untargeted metabolomic analysis under different storage conditions after 180 days of storage. The purpose of this study is to elucidate the effects of different storage conditions on high moisture rice and to seek a method that favors the storage of high moisture rice, providing a theoretical foundation for the proper storage of high moisture rice.

## 2. Materials and Methods

### 2.1. Materials and Reagents

In November 2022, rice with the variety of “Huanghuazhan” was harvested in Wuhan, China. Rice was then dried to maintain a moisture content of 15.5 ± 0.5%. Anhydrous ethanol and phenolphthalein were of analytical grade and were obtained from Komeo (Tianjin Komeo Chemical Reagent Co., Ltd., Tianjin, China). Trichloroacetic acid (TCA), thiobarbituric acid (TBA), 95% ethanol, acetic acid, iodine, potassium iodide, and potassium hydroxide were of analytical grade and were obtained from Aladdin (Shanghai Aladdin Biochemical Technology Co., Ltd., Shanghai, China). Ammonium formate, ammonia, and formic acid were chromatographically pure and purchased from Aladdin (Shanghai Aladdin Biochemical Technology Co., Ltd., Shanghai, China). Methanol and acetonitrile were chromatographically pure and purchased from Sigma-Aldrich (Sigma Chemical Co., St. Louis, MO, USA). Chromatographically pure acetic acid was purchased from Rhawn (Shanghai Eon Chemical Technology Co., Ltd., Shanghai, China). Chemical standard products of analytical grade were purchased from Sigma (Sigma Chemical Co., St. Louis, MO, USA).

### 2.2. Storage

Two groups were used in the experiment. CS: rice was placed in 0.24 mm polyethylene bags, and it was stored in a chamber with a constant temperature of 30 ± 1 °C and humidity of 65 ± 2%. LT + MA: rice was placed in 0.24 mm polyethylene bags, and nitrogen was added to maintain a concentration of 95 ± 0.1%. These samples were then stored in a chamber with a constant temperature of 20 ± 1 °C and humidity of 65 ± 2%.

### 2.3. Fatty Acid Value

The FAVs were determined in accordance with the methodology proposed by Qu et al. [12]. The rice samples were dehulled using a dehulling machine (THU35C, Satake Machinery (Suzhou) Co., Ltd., Suzhou, China) and were pulverized using the mill (TDW-5000, Beijing Tongxin Tianbo Technology Development Co., Ltd., Beijing, China). 10 g of rice powder and 25 mL of anhydrous ethanol were mixed in a 250 mL stoppered conical flask. Vigorously agitated, set aside, and then filtered to extract the fat. After filtration, phenolphthalein was used as an indicator, and potassium hydroxide-95% ethanol solution (0.01 mol/L KOH) was titrated. The quantity of solution consumed was recorded to calculate FAV, which represents the mg of KOH consumed to neutralize fatty acids in 100 g of powder (mg/100 g).

### 2.4. Malondialdehyde Content

The method of Li et al. [13] was adopted with minor modifications. A 1.00 g sample of dehulled rice powder was mixed with 5 mL of 10% trichloroacetic acid (TCA). This mixture was then centrifuged at 10,000× *g* for 15 min at 4 °C. Then, the supernatant was filtered and fixed to 7 mL. 2 mL of this solution was aspirated and mixed with 2 mL of 0.67 % thiobarbituric acid (TBA) and boiled in a water bath for 15 min, followed by rapid cooling. MDA content was determined by absorbance at wavelengths 450 nm, 532 nm, and 600 nm using a UV-visible spectrophotometer (T700, PGeneral, Beijing, China) and expressed as mg/100 kg.

### 2.5. Amylose Content

The amylose content was analyzed in accordance with the methodology proposed by Qu et al. [12]. The rice samples were milled and ground to produce milled rice powder, sieved through a 100-mesh sieve, and defatted using petroleum ether reflux. The milled rice powder was left in a fume hood for 24 h, then refrigerated at 4 °C. A 100 mg ± 0.5 mg sample was transferred to a 100 mL conical flask, mixed with 1 mL of 95% ethanol and 9.0 mL of 1.0 mol/L NaOH, and boiled for 10 min to disperse the starch. After cooling, the solution was diluted to 100 mL. 5 mL of the test solution was pipetted and mixed with 50 mL of distilled water, 1.0 mL of 1 mol/L acetic acid, and 2.0 mL of iodine reagent (prepared by dissolving 2.0 g of potassium iodide and 0.2 g of iodine in 100 mL of distilled water) in a 100 mL flask. The blank used 5 mL of 0.09 mol/L NaOH. The absorbance of the solution was measured at a wavelength of 720 nm using a UV-visible spectrophotometer (T700, PGeneral, Beijing, China).

### 2.6. Rice Color Measurement

The color of the rice was quantified using a colorimeter (CR-410, KONICA MINOLTA, Tokyo, Japan), which was initially calibrated with a whiteboard. Thereafter, the color of the rice was measured and recorded as L* (lightness), a* (red-green), and b* (yellow-blue).

### 2.7. Pasting Properties of Rice

Pasting properties of rice were tested using a Rapid Viscosity Analyzer (RVA) instrument (RVA-TecMaster, Perten Instruments, Inc, Stockholm, Sweden) based on the methods of Chen et al. [14] The samples were prepared by adding 25.00 ± 0.10 mL of water and 3.00 ± 0.01 g of rice flour to a dry and clean sample jar. The sample was transferred to the sample brief, the stirrer placed within the sample brief, and the specimen dispersed by rapid up-and-down stirring for 10 times. The experiment was carried out according to the warming program set by the experimental method. Meanwhile, the drivers and the pasting properties were recorded, and the RVA curves were reported in cP or °C units.

### 2.8. Scanning Electron Microscopy (SEM)

The rice samples were analyzed using a Sigma 300 scanning electron microscope (Carl Zeiss AG, Baden-Württemberg, Germany). The samples were sectioned at one-third and two-thirds of the rice using liquid nitrogen to observe the cross-section of the rice [15]. The samples were fixed on a sample holder with an aluminum plate, and postfixation was coated with platinum (Pt) in a vacuum evaporator. SEM images were obtained under high vacuum conditions using an accelerating voltage of 3 kV. The secondary electron images were examined at varying magnifications.

### 2.9. UHPLC-TOF-MS Conditions for Metabolomics Analysis

#### 2.9.1. Metabolite Extraction

The rice samples were taken after the 180-day storage period. Samples were promptly placed in a −80 °C refrigerator until assayed. The rice samples were submitted to freeze-drying in a lyophilizer (Scientz-100F), after which the samples were pulverized into a powdered form using a grinder (30 Hz, 1.5 min). A total of 50 mg of the sample was then transferred to a centrifuge tube, followed by the introduction of 1200 µL of extraction solution (pre-cooled 70% methanol in water at −20 °C). Vortexing every 30 min for 30 s for a total of 6 vortexes. After centrifugation (rotation speed 13,523× *g*, 3 min), the samples were filtered through a microporous filter membrane with a pore size of 0.22 μm. The supernatant was then collected for injection into a plastic bottle autosampler. All samples were combined with an equal volume of supernatant to create a quality control (QC) sample for analysis.

#### 2.9.2. UHPLC-TOF-MS Analysis

Non-target metabolomic analysis of rice samples was performed at the end of storage. UHPLC-TOF-MS analysis was conducted using an ultra-high performance liquid chromatography system (LC-30A, Shimadzu, Japan) with a Waters ACQUITY Premier HSS T3 Column (2.1 mm × 100 mm, 1.8 μm) and mass spectrometer (TripleTOF 6600+, SCIEX, Framingham, MA, USA). The mobile phase A: 0.1% formic acid in water; mobile phase B: 0.1% formic acid in acetonitrile. The autosampling volume is 4 μL, the flow rate is 0.4 mL/min, and the temperature is 40 °C.

The data acquisition was conducted using information-dependent acquisition (IDA) mode with Analyst TF 1.7.1 software (Sciex, Concord, ON, Canada). The ESI source conditions were set as follows: ion source gas 1 (GAS1), 50 psi; ion source gas 2 (GAS2), 50 psi; curtain gas (CUR), 25 psi; temperature (TEM), 550 °C; declustering potential (DP), 60 V or −60 V in positive or negative modes, respectively; and ion spray voltage-floating (ISVF), 5000 V or −4000 V in positive or negative modes, respectively. The unprocessed data were converted to the mzXML format using ProteoWizard and subsequently treated with further processing using an in-house program developed using R and based on XCMS. This program was used for peak detection, extraction, alignment, and integration. The corrected and screened peaks were employed for the purpose of metabolite identification.

### 2.10. Statistical Analysis

The experimental data were evaluated using a one-way ANOVA and Duncan’s Multiple Extreme Variance Test using SPSS (version 20.0, SPSS Inc., Armonk, NY, USA). Data were expressed as the means ± standard deviation of three replicates; a *p*-value < 0.05 is considered statistically significant, and the letters abcd are used in place of * to indicate the significance of differences.

The annotation of metabolites was conducted using the online databases Kyoto Encyclopedia of Genes and Genomes (KEGG) (https://www.genome.jp/kegg/pathway.html, accessed on 17 January 2025) and Human Metabolome Database (HMDB) (https://hmdb.ca/metabolites, accessed on 17 January 2025). Metware Cloud (https://cloud.metware.cn, accessed on 17 January 2025) was utilized to conduct the PCA, OPLS-DA, volcano plots, heatmap of hierarchical clustering analysis (HCA), and metabolic pathways analysis maps, in addition to pathway annotation maps.

## 3. Results and Discussion

### 3.1. Changes in Rice Quality

#### 3.1.1. Fatty Acid Values

**Figure 1:**
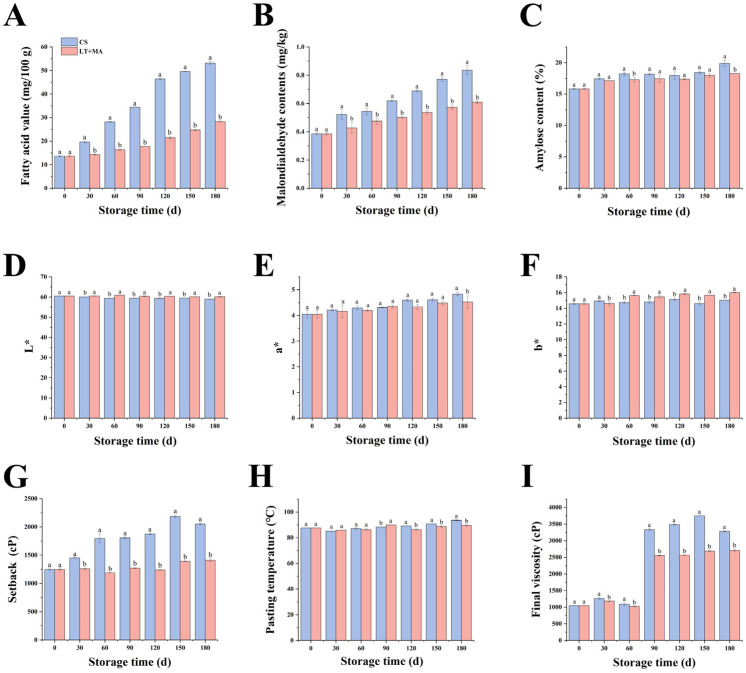
Changes in quality characteristics of rice during storage under two storage conditions, LT + MA (low temperatures, nitrogen, and modified atmosphere) and CS (conventional storage). (**A**) Changes of fatty acid value (FAV) for LT + MA and CS, (**B**) Changes of malondialdehyde (MAD) content for LT + MA and CS, (**C**) Changes of amylose content for LT + MA and CS, (**D**–**F**) Changes of color for LT + MA and CS, (**G**–**I**) Changes of pasting properties for LT + MA and CS. A *p*-value of less than 0.05 was considered statistically significant, and the letter ab is employed instead of * to denote the significance of the difference.

FAV were key indicators for assessing rice deterioration during storage [16]. The production of free fatty acids from stored fat was slower than the degradation of free fatty acids, which reduces FAV during storage. Moreover, the presence of free fatty acids in stored rice causes physicochemical changes, including oxidation and hydrolysis, which result in the degradation of rice quality and the loss of nutrients. Figure 1A illustrates FAV changes in rice with different storage conditions during storage. The FAVs increased under the two storage conditions, with significant differences in FAVs observed after 30 days of storage. Furthermore, at the end of the storage period, FAV in the LT + MA group was 53.33% of that in the CS group, which was significantly lower than that under CS storage (*p* < 0.05), thereby confirming that LT + MA effectively slowed down the rate of fat oxidation. Liu et al. [16] demonstrated that the temperature during storage of rice was positively correlated with FAV. Furthermore, Qu et al. [12] showed that a nitrogen-modified atmosphere inhibited the increase of FAV. The results of this study are consistent with their findings of the study. Consequently, lipolysis was suppressed under LT + MA storage conditions, thereby ensuring the maintenance of quality in high moisture rice.

#### 3.1.2. Malondialdehyde Content

MDA, a carbonyl byproduct of oxidative damage, serves as an indicator of lipid oxidation in rice. MDA has been observed to cross-link with lipids and proteins in the cell membrane, resulting in a loss of membrane fluidity and integrity and consequent abnormal cell membrane permeability [17]. Consequently, this can result in a range of cellular dysfunctions, including mitochondrial dysfunction, ion homeostasis imbalance, and even apoptosis or necrosis. MDA content has been reported to increase with longer storage time or accelerated aging [18]. As shown in Figure 1B, the MDA content gradually increased with the extension of the storage period. At the end of the storage period, the MDA content in the LT + MA group was 72.93% of that in the CS group, which was significantly lower than that under CS storage (*p* < 0.05). The results demonstrated that LT + MA retarded the degree of lipid oxidation in rice, effectively mitigated oxidative damage, improved rice quality, and extended the storage period.

#### 3.1.3. Amylose Content

The primary constituent of rice is starch, which can account for up to 90% of the total dry matter of refined rice [19]. Rice starch was included in amylopectin and amylose. The low amylose content enhances the favorable eating quality of cooked rice [20]. As shown in Figure 1C, the amylose content of rice increased with the duration of storage. The rise in the amount of amylose present in rice treated with LT + MA was significantly impeded compared to that observed in CS. The content of amylose content after 180 days of storage increased from the initial 15.82% to LT + MA (18.27%) and CS (19.89%) groups, respectively. The LT + MA group was 0.92 times greater than the CS group. The texture of cooked rice was primarily determined by the amylose content of rice kernels. The lower the amylose content in the rice, the softer the texture of the cooked rice [21]. Thus, LT + MA treatment is helpful for the edible quality maintenance of rice.

#### 3.1.4. Color

There was a robust correlation between the color of the rice seed coat and its quality, with yellowing of the rice seed coat being a hallmark of deterioration in quality. As shown in Figure 1D, the brightness (L*) value of rice decreases and rice becomes darker as the storage time increases. At the end of storage, the L* of the LT + MA group was 1.04 times higher than that of the CS group. The brightness of rice in the LT + MA group was significantly higher than that of the CS group. In addition, only after 180 days did the a* values of rice in the CS group (4.830) differ significantly from the LT + MA group (4.525). The b* values produced significant differences after 60 days, and b* in the LT + MA group was 1.07 times higher than that in the CS group after 180 days of storage. LT + MA treatment reduced b* values and increased L* values. LT + MA was able to better suppress the reduction of the brightness of the rice, thus delaying the aging of rice. LT + MA treatment effectively reduced the elevation of FAV in FAV experiments, and LT + MA treatment reduced the b* value in color experiments. Jang et al. [22] showed that the b* value of rice was positively correlated with FAV. This is consistent with our findings, and in summary, LT + MA effectively suppressed the deterioration of rice quality.

#### 3.1.5. Pasting Properties

The pasting properties exert a significant influence on the processing of rice. Final viscosity, gelatinization temperature, and setback value can be obtained by measuring the pasting properties of rice [15]. Setback reflects the degree of aging of the sample at low temperatures and the stability of the cold paste [23]. Wang et al.’s [24] study indicated that a certain degree of regeneration has a positive effect, while setback was significantly negatively correlated with the taste value. With the prolongation of storage time, the setback of rice fluctuated upwards but increased more slowly under LT + MA conditions (Figure 1G). This indicates that LT + MA reduces the degree of aging of rice, enhances the stability of the rice, and improves the taste value of the rice.

The pasting temperature is the critical temperature where rice starch granules in an aqueous solution absorb water through heat, leading to irreversible swelling. This process results in the destruction of the natural crystal structure of the granules and the disappearance of birefringence [25]. The pasting temperature has an impact on the volumetric expansion, water absorption, and softness of rice during the cooking process [26]. The pasting temperature displays a fluctuating and rising trend with increasing storage periods (Figure 1H). The trend of increasing pasting temperature from 120 days onwards in the LT + MA group was significantly lower than in the CS group. This finding suggests that LT + MA rice was more readily pasteable than CS rice. In general, rice starch with a high content of amylose has a higher pasting temperature and a higher setback viscosity [27], which was consistent with the findings of the present study.

The final viscosity indicates the ability of the starch paste to form a gel and paste after cooling. This may reflect the regeneration ability of the starch. In general, a larger final viscosity corresponds to a harder rice and a worse taste [28]. The application of LT + MA has been demonstrated to impede the deterioration of rice. The final viscosity of LT + MA was reduced, resulting in a softer texture and improved eating quality of the rice after cooking and cooling. The content of amylose exerts a significant influence on the final viscosity of starch pasting. In general, the content of amylose was positively proportional to the final viscosity [29]. This study found that LT + MA inhibits the increase in final viscosity and thereby enhances the texture of rice.

#### 3.1.6. SEM

**Figure 2:**
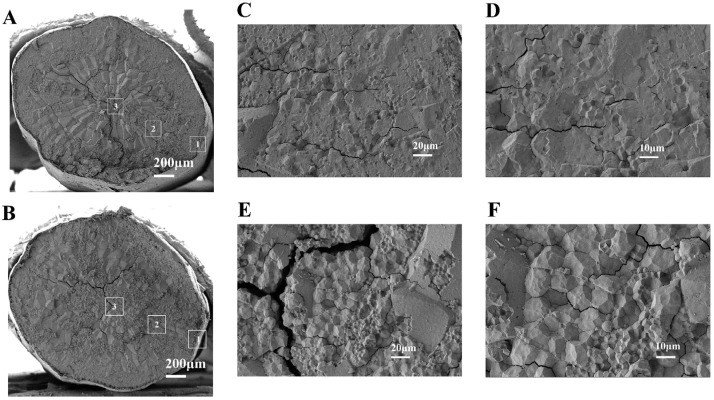
SEM images of cross-sections of raw rice kernels for CS and LT + MA. Cross-section of the entire rice kernel for CS (**A**) and LT + MA (**B**). (**C**) (×500) and (**D**) (×1000) are different magnifications of A, respectively. (**E**) (×500) and (**F**) (×1000) are different magnifications of B, respectively. In (**A**,**B**), 1 is the testa tissue, 2 is the aleurone layer, and 3 is the central area of the starchy endosperm.

SEM images of cross-section were employed to elucidate the microstructure characteristics of the rice. Figure 2A,B illustrated the entire cross-section of raw rice kernels after 180 days of storage for both the CS and LT + MA groups of rice. The presence of testa tissue (Figure 2A(1)), the aleurone layer (Figure 2A(2)), and central areas (Figure 2A(3)) of the starchy endosperm were depicted in Figure 2A,B. Normal starch granules present a consistent polygonal shape. In contrast, when malignant whitening occurs with rice aging, the edges of the chalky portion of the starch granules are not obvious [14]. As illustrated in Figure 2, the polygonal pattern of the CS storage rice starch granules was not obvious. In contrast, the starch granules in the LT + MA storage rice samples exhibited a composite structure with clear edges of a regular polygon pattern. The LT + MA storage (Figure 2E,F) method was observed to be more effective than CS storage (Figure 2C,D) in maintaining the regular polygonal pattern and having a distinct edge of rice.

### 3.2. Metabolome Profiling of LT + MA vs. CS

#### 3.2.1. Multivariate Statistical Analysis

In the experiment, UHPLC-TOF-MS was used to analyze the rice samples in both positive ion (POS) mode and negative ion (NEG) mode, and a total of 776 metabolites were detected in the NEG mode, while 939 metabolites were detected in the POS mode.

**Figure 3:**
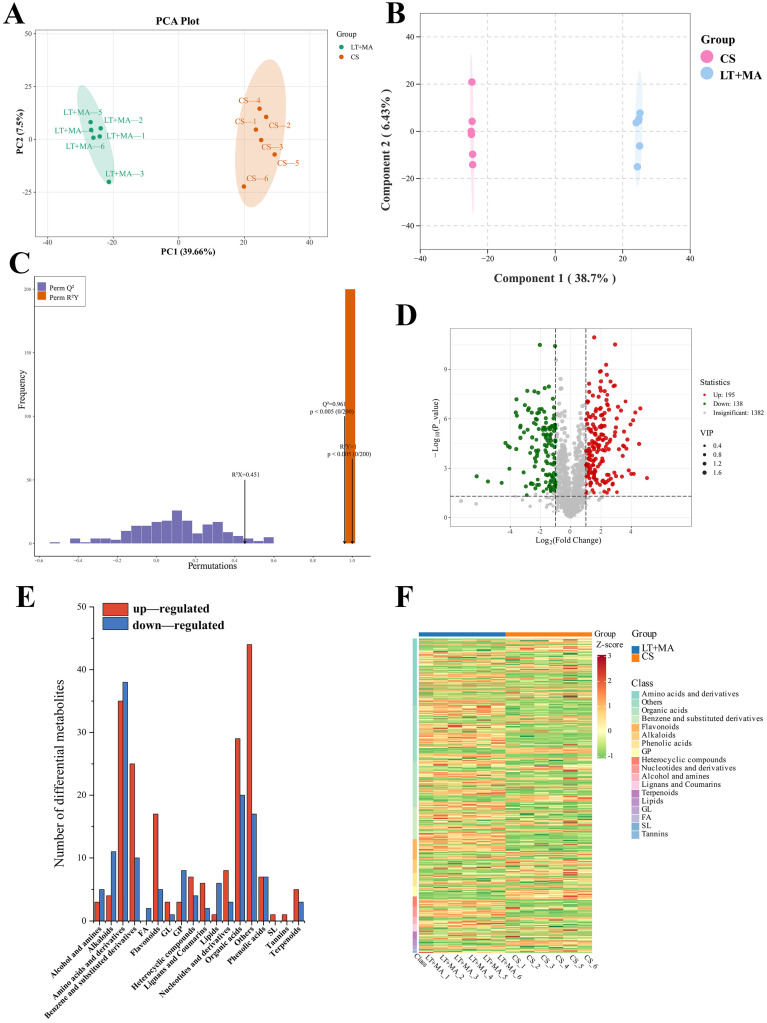
(**A**) PCA score plots of CS and LT + MA, (**B**) OPLS-DA score plots of CS and LT + MA, (**C**) Permutation histogram test of OPLS-DA model. (**D**) Volcano plot for group CS vs. LT + MA. (**E**) Classification of the differential metabolites in LT + MA vs. CS. The red and blue colors represent up-regulated and down-regulated metabolites, respectively. The numbers above the bar are the amounts of the metabolites. (**F**) Heatmap of hierarchical clustering analysis for group CS vs. LT + MA.

In order to gain an understanding of the metabolic difference between the samples, the detected metabolites were subjected to a principal component analysis (PCA). Figure 3A shows that the first principal component (PC1) and the second principal component (PC2) explained 39.66% and 7.5% of the total variance, respectively. The results showed that the two groups showed a trend of separation at the level of PC1. To distinguish the different groups and obtain clearer separation results, a supervised OPLS-DA model was used (Figure 3B). The metabolites of rice stored under different conditions were clearly distinguished from each other, as evidenced by the OPLS-DA model. A greater demonstration of between-group differences can be represented by OPLS-DA. However, supervised classification models have the disadvantage that overfitting may occur. Consequently, in the case of supervised classification models, it is necessary to verify the reliability of the validation model (Figure 3C). A total of 200 permutation tests were conducted on the experimental data, which yielded the following results: Q2 = 0.961, R2Y = 1, *p* < 0.005. R2 denotes the explanatory power of the model, and Q2 denotes the predictive power of the model. The Q2 values in models with a value greater than 0.5 indicated a satisfactory effectiveness model. The score plots demonstrate clear separation and differentiation, which is indicative of an accurate and reliable model.

The Fold Change and VIP values were employed to identify differential metabolites, with metabolites that satisfied both the Fold Change (FC) > 2 and VIP > 1 criteria being considered as such. The up- and down-regulation of differential metabolites can be represented by volcano plots (Figure 3D), each point representing one metabolite, where red represents up-regulation, green represents down-regulation, and grey represents no significant change. The results indicated that 195 metabolites were up-regulated and 138 down-regulated for LT + MA vs. CS.

Differential metabolites of LT + MA vs. CS include organic acids, amino acids and derivatives, benzene and substituted derivatives, saccharides, flavones, and flavonols, among others (Table S1) (Figure 3E). CS vs. LT + MA, the most abundant of which was amino acids and derivatives, totaling 73 species, and a total of 24 glycan differential metabolites were present, of which only 2-O-alpha-L-rhamnopyranosyl-D-glucopyranose, N,N′,N″-triacetylchitotriose, D-fructose, and acetyl-maltose occurred down-regulated, and the rest of the differential metabolites were up-regulated. A total of 45 bioactives, including terpenoids, alkaloids, and flavonoids, yielded the occurrence of differential metabolites, of which only four differential metabolites—dopamine, arecaidine, N-feruloyl dopamine, and trans- and DIMBOA glucoside—were up-regulated out of a total of 15 alkaloids.

The Euclidean distance matrix was generated using the quantitative values of differential metabolites of rice with varying moisture content. Differential metabolites were clustered using a complete chaining method and presented in a heatmap of hierarchical clustering. The vertical coordinates represent the differential metabolites of a given group comparison. The color blocks at different positions represent the relative expression of the metabolites at those corresponding positions. The color red indicates that the substance in question was expressed at a high level within the group, while the color green indicates that the substance was expressed at a low level within the group. Changes in the different kinds of metabolites can be seen in Figure 3F. The differential metabolites of LT + MA vs. CS were primarily alkaloids, benzene and substituted derivatives, phenolic acids, flavonoids, organic acids, amino acids and derivatives, and saccharides.

#### 3.2.2. KEGG Enrichment Analysis of Differential Metabolites

**Figure 4:**
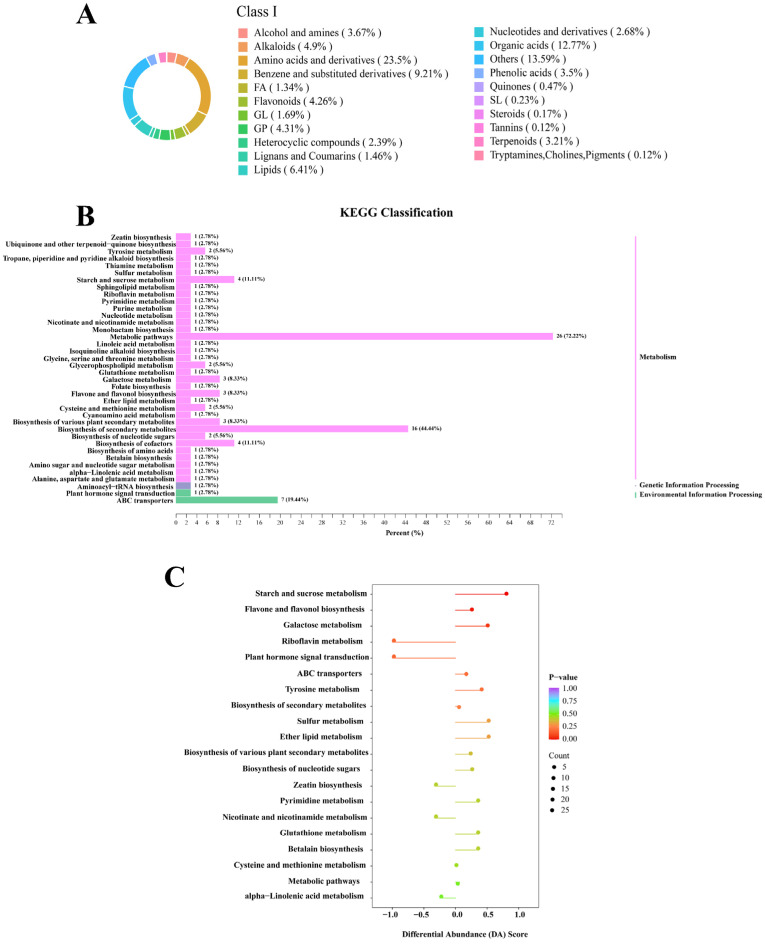
(**A**) Classification of overlapping differential metabolites in rice under two storage conditions. (**B**) The vertical coordinate represents the name of the metabolic pathway, while the horizontal coordinate denotes the number of differential metabolites that have been annotated to the pathway, as well as the ratio of these metabolites to the total number of differential metabolites to which they have been annotated. (**C**) The horizontal axis denotes the corresponding Rich Factor of each pathway, the vertical axis represents the pathway name (sorted by *p*-value), and the color of the dots reflects the size of the *p*-value, with redder hues indicating greater significance in pathway enrichment. The color of the dot is indicative of the size of the *p*-value, with redder dots denoting a greater degree of significant enrichment. The size of the dot is indicative of the number of differentially enriched metabolites. The horizontal coordinate is the log FC (log-fold change) of the differential metabolite. This can be expressed as the logarithmically transformed, base 2-fold change value for the differential metabolite in question.

Figure 4A shows the composition of metabolite classes identified by UHPLC-TOF-MS; the different classification categories were represented by different color blocks, with the percentages indicating the proportion of metabolites belonging to each category out of the total number of metabolites identified.

In order to gain further insight into the differential pathways present at the storage endpoint of rice, an enrichment analysis was performed using the KEGG database. The results of the metabolic pathway analysis are presented in the form of bubble plots, wherein each bubble represents an identified metabolic pathway. Figure 4B illustrates the percentage of differential metabolites in each pathway. Figure 4C identified the top 20 enriched metabolic pathways, revealing high enrichment in starch and sucrose metabolism and flavone and flavonol biosynthesis pathways after 180 days of storage.

#### 3.2.3. Differential Metabolites Analysis

**Figure 5:**
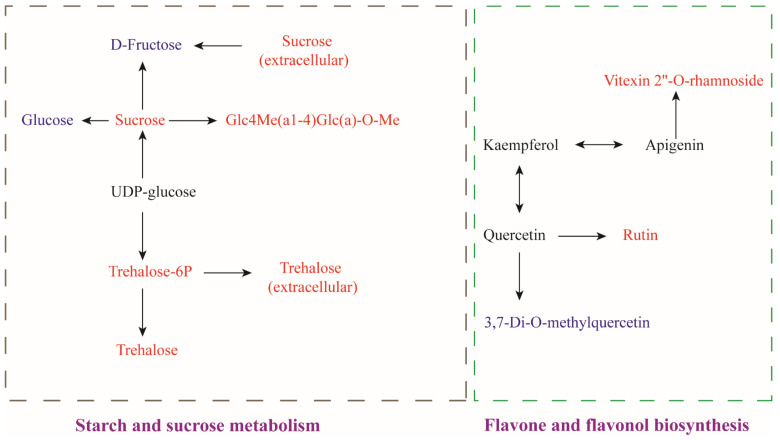
Metabolites interrelationship map. The red font indicates a higher expression of the substance than CS in the LT + MA group, while the blue font denotes a lower expression of the substance than CS in the LT + MA group. The black font is used to represent no change between the two groups.

The differential metabolites were assessed at the end of the storage and pathway analysis, thereby revealing the physiological mechanisms that produce different changes during the storage of rice under different conditions. The metabolic synthesis pathways were depicted in Figure 5.

##### Starch and Sucrose Metabolism

At the end of the storage period, the expression levels of key metabolites such as sucrose, trehalose-6-phosphate (T6P), alginate, and Glc4Me(a1-4)Glc(a)-0-Me in the starch and sucrose metabolism pathways were significantly up-regulated in the LT + MA group as compared to the CS group.

Sucrose is the principal source of carbon for growth, development, and defense. Sucrose synthesis was catalyzed by sucrose phosphate synthetase (SPS) and sucrose phosphatase (SPP), with sucrose 6-phosphate serving as an intermediate. SPS is a pivotal regulatory enzyme within the sucrose synthesis pathway. As storage time increases, sucrose is hydrolyzed to glucose and fructose by enzymes, a process that leads to a gradual decrease in sucrose content [30]. Low temperature treatment significantly increased SPS activity. Thus, sucrose catabolism was inhibited under LT + MA storage conditions, resulting in a significant increase in sucrose content. Sucrose metabolism is integral to the production of sugar transporter molecules that are essential for plant abiotic stress tolerance and sensitivity [31]. It was observed that the sucrose content of LT + MA storage rice was significantly increased compared to CS storage, which indicated that LT + MA storage exerted a significant inhibitory effect on excessive sucrose consumption. These findings indicate that LT + MA effectively delays the excessive depletion of sucrose, enhances the environmental tolerance of rice seeds, and extends their shelf life.

Trehalose-6-phosphate (T6P) was synthesized from UDP-glucose and glucose-6-phosphate (G6P) by trehalose-6-phosphate synthase (TPS). T6P was dephosphorylated by trehalose-6-phosphate phosphatase (TPP) to produce trehalose. TPP is the enzyme that controls the final step of trehalose biosynthesis. Changes in TPS and TPP activity were highly consistent. Finally, trehalose was hydrolyzed into two molecules of glucose by trehalase. The activity of TPS is enhanced at low temperatures, which promotes the production of T6P [32]. At the same time, TPP activity was enhanced, promoting trehalose synthesis. In comparison with CS, the content levels of trehalose-6-phosphate (T6P) and trehalose were significantly elevated in rice treated with LT + MA treatment, as demonstrated in Figure 5. It has been shown that the ratio of T6P content to sucrose content in plants affects a number of plant physiological activities such as starch synthesis. In certain yeast species, the accumulation of T6P was essential for maintaining cellular integrity [33]. Trehalose plays a pivotal role in cellular protection during stress in all organisms. It serves as an energy source, a storage and transport molecule for glucose, and a stress-responsive compound. In addition, trehalose was also a stabilizer and protector of membranes and proteins and could prevent oxygen radical damage and regulate the glycolytic pathway [34]. Simultaneously, trehalose contributes to maintaining cellular integrity. In our experiments, the MDA content increased over time. The trend of increase in the LT + MA group was less pronounced than that in the CS group, indicating that LT + MA was effective in maintaining the integrity of the cell membrane. In our study, LT + MA treatment had elevated trehalose and T6P content, and high levels of trehalose and T6P promoted cellular resistance to the environment, enhanced rice quality, and prolonged the storage period.

##### Flavone and Flavonol Biosynthesis

Compared to the CS treatment, the LT + MA treatment demonstrated a down-regulation in 3,7-Di-O-methylquercetin (DMQ) and an up-regulation in rutin and Vitexin 2″-O-rhamnoside (VOR) in the biosynthetic pathway of flavonoids and flavonols. Previous studies indicated that flavonols regulate key developmental processes in eukaryotic cells under environmentally altered cellular redox homeostasis [35].

Rutin is one of the naturally occurring flavonoids in plants. It was a glycoside formed by dehydration of the flavonol glycoside quercetin and a disaccharide composed of rhamnose and glucose [36]. Rutin is a potent natural antioxidant that has been demonstrated to reduce oxidative stress [37]. The phenolic hydroxyl group in rutin donates hydrogen atoms or electrons, neutralizing free radicals and impeding free radical chain reactions. The phenoxyl free radicals that were formed when rutin itself is oxidized were relatively stable and can evade further triggering of oxidation reactions [38], thereby preventing structural damage to biomolecules and biofilms from the excess free radicals generated by stress or abnormal metabolic processes. It has been demonstrated that MDA content can be indicative of a positive correlation between the degree of lipid peroxidation and the degree of adversity injury suffered by plants [17]. The LT + MA group treatment was observed to have a slowing effect on the rate of increase of MDA content and a reduction in the degree of peroxidation in plants. In contrast to the CS group, the content of rutin increased in the LT + MA treatment group, thereby attenuating the effects of the rice on the cells due to oxidative stress suffered and contributing to the protection of the integrity of the cell membrane of the rice.

The conversion of quercetin to rutin occurs through a continuous two-step glycosylation reaction, which is catalyzed by a specific glycosyltransferase. The mechanism of conversion of 3,7-Di-O-methylquercetin (DMQ) to quercetin mainly involves demethylation reactions. In this, DMQ acts as a substrate and removes the methyl groups at its 3-position and 7-position in the presence of specific enzymes, thus reverting to quercetin. LT + MA promoted the conversion of quercetin to rutin and DMQ to quercetin.

Vitexin-2″-O-rhamnoside (VOR) has been demonstrated to possess an inhibitory effect on lipid peroxidation. In addition, it exhibits a high hydroxyl radical scavenging rate [39]. VOR has been shown to have high activity in scavenging reactive oxygen species (ROS) in cells, attributable to the carbon 2, 3-double bond on the B-ring of the VOR structure. This may extend the conjugated system and significantly protect cells against H_2_O_2_-induced damage [40]. In our experiments, the LT + MA group exhibited reduced MDA levels and decreased oxidative damage to the cells, and the VOR content of rice treated with LT + MA was higher than that of rice treated with CS after storage, indicating that the LT + MA combination had better antioxidant properties and retained the quality of rice to a greater extent. As a result, flavone and flavonol biosynthesis regulated the antioxidant properties of the rice. The production of DMQ was reduced, and the production of rutin was promoted under LT + MA conditions. The up-regulation of rutin and VOR under LT + MA conditions reduced the damage due to oxidative stress in rice, which helped to protect rice cell membrane integrity.

The LT + MA treatment mainly affects carbohydrate metabolism and the biosynthesis of flavonoids and flavonols compared to CS. It prolongs higher moisture rice storage by inhibiting grain quality deterioration through enhanced antioxidants within the grains.

## 4. Conclusions

This study investigated the effects of combined low temperature and nitrogen-modified atmosphere (LT + MA) treatments on high moisture rice storage quality, microstructure, and metabolites. LT + MA treatment effectively reduced the FAV, MDA content, and amylose content of rice. LT + MA treatment inhibited the reduction of L* and b*, inhibited the deterioration of the pasting characteristics of rice, and maintained the polygonal structure of rice. Moreover, LT + MA treatment delayed the aging of rice and enhanced grain vigor by modulating the starch and sucrose metabolism pathways, as well as the flavonoid and flavonol biosynthesis pathways. Thus, LT + MA treatment is an effective preservation technology in retarding quality deterioration of high moisture rice during storage.

## Data Availability

The original contributions presented in the study are included in the article/Supplementary Materials; further inquiries can be directed to the corresponding author.

## Supplementary Materials

The following supporting information can be downloaded at https://www.mdpi.com/article/10.3390/foods14071262/s1: Table S1. Significantly changed metabolites between the conventional storage (CS) and nitrogen modified atmosphere and low temperatures (MA + LT) by the ANOVA test analysis and OPLS-DA analysis.
