# Supplementary material for: Effect of Low Temperature and Nitrogen Modified Atmosphere Treatments on the Storage of High Moisture Indica Rice: Quality, Microstructure, and Metabolome Characteristics
Source: Foods. 2025 Apr 3;14(7):1262. doi: 10.3390/foods14071262 (PMC11988486; doi:10.3390/foods14071262)
Supplement: Supplementary file 1 [file foods-14-01262-s001.zip › foods-3517079-supplementary.pdf]

Table S1 Significantly changed metabolites between the conventional storage (CS) and nitrogen modified atmosphere and low temperatures (MA+LT) by the ANOVA test analysis and OPLS-DA analysis.

Compounds	Class	Molecule Formula	VIP	*p*-value	DOWN
omega-Aminoarginine	amino acids and derivatives	C6H15N5O2	1.51	2.00E-08	down
Phe-Gly	amino acids and derivatives	C11H14N2O3	1.45	9.10E-03	down
Asparagine	amino acids and derivatives	C4H8N2O3	1.04	1.95E-03	up
Nα-Acetyl-L-Arginine	amino acids and derivatives	C8H16N4O3	1.51	4.23E-07	down
1-[(5-Amino-5-carboxypentyl)amino]-1-deoxyfructose	amino acids and derivatives	C12H24N2O7	1.56	3.91E-04	down
N2-Fructopyranosylarginine	amino acids and derivatives	C12H24N4O7	1.53	6.54E-08	down
Ureidosuccinic acid	amino acids and derivatives	C5H8N2O5	1.60	4.42E-07	up
2-Octanamidoacetic acid	amino acids and derivatives	C10H19NO3	1.49	1.78E-03	down
Gly-Gly-Phe	amino acids and derivatives	C13H17N3O4	1.57	5.29E-05	down
Succinylproline	amino acids and derivatives	C9H13NO5	1.45	6.69E-06	down
Glutaminylphenylalanine	amino acids and derivatives	C14H19N3O4	1.21	4.29E-02	down
Tyr-Pro-Ile	amino acids and derivatives	C20H29N3O5	1.24	1.41E-02	down
Pro-Leu-Tyr	amino acids and derivatives	C20H29N3O5	1.38	1.01E-05	down
Pro-Tyr-Val	amino acids and derivatives	C19H27N3O5	1.21	1.05E-02	down
Leu-Nap-OH	amino acids and derivatives	C24H24N2O6	1.12	6.92E-02	up
His-Asn-Ala-Glu	amino acids and derivatives	C18H27N7O8	1.26	8.81E-08	up
Ser-Ile-His-Arg	amino acids and derivatives	C21H37N9O6	1.38	1.27E-02	down
(2R)-3-(5-acetamido-2-hydroxyphenyl)sulfanyl-2-azaniumylpropanoate	amino acids and derivatives	C11H14N2O4S	1.47	1.69E-04	up
Pro-Ser-Asp	amino acids and derivatives	C12H19N3O7	1.48	2.56E-07	down
Tyr-Asp-Ser	amino acids and derivatives	C16H21N3O8	1.37	9.70E-04	up
Leu-Arg-Gly-Gln-Asp	amino acids and derivatives	C23H41N9O9	1.09	7.84E-05	down
Arg-Leu-Val-Glu	amino acids and derivatives	C22H41N7O7	1.30	3.71E-03	down
TyrMe-Lys-OH	amino acids and derivatives	C22H27N3O7	1.56	7.80E-04	down
Glu-Pro-His	amino acids and derivatives	C16H23N5O6	1.38	1.28E-04	up
Glu-Glu-Leu-Ile-Thr	amino acids and derivatives	C26H45N5O11	1.55	6.91E-05	up
Asp-Phe-Ser	amino acids and derivatives	C16H21N3O7	1.37	8.51E-05	down
Glutathione Reducedform	amino acids and derivatives	C10H17N3O6S	1.48	4.64E-05	up
Ala-Ser-Asn-Asp-Leu	amino acids and derivatives	C20H34N6O10	1.06	2.34E-02	down
Tyr-Cys	amino acids and derivatives	C12H16N2O4S	1.27	1.26E-03	up
Glu-Gln-Ile	amino acids and derivatives	C16H28N4O7	1.40	9.84E-02	down
Tyr-Glu-Arg-Arg	amino acids and derivatives	C26H42N10O8	1.48	2.63E-06	down
Asn-Ser-Glu-Asp-Thr	amino acids and derivatives	C20H32N6O13	1.39	8.50E-03	up
Met-Ser-Asn	amino acids and derivatives	C12H22N4O6S1	1.45	1.27E-02	down
L-Histidine,L-lysyl-	amino acids and derivatives	C12H21N5O3	1.39	2.48E-04	up
Glu-Asp-Thr-Glu	amino acids and derivatives	C18H28N4O12	1.57	1.10E-08	down
Cys-Thr-Val	amino acids and derivatives	C12H23N3O5S1	1.50	1.02E-04	up
Ile-Arg-Ile-Ile-Val	amino acids and derivatives	C29H56N8O6	1.58	2.89E-05	down
Tyr-Glu-Arg	amino acids and derivatives	C20H30N6O7	1.57	3.96E-05	up
Gln-Gln-Gln	amino acids and derivatives	C15H26N6O7	1.57	2.64E-05	up
Phe-Glu-His-Asp	amino acids and derivatives	C24H30N6O9	1.57	1.37E-09	up
Val-Val-Asn-Trp-Asp	amino acids and derivatives	C29H41N7O9	1.49	7.26E-07	up
Met-Phe-Thr-Glu-Asp	amino acids and derivatives	C27H39N5O11S	1.46	9.34E-05	up
Asp-Ser	amino acids and derivatives	C7H12N2O6	1.40	2.35E-03	up
Asp-Pro-Ser	amino acids and derivatives	C12H19N3O7	1.25	4.87E-03	down

Leu-Cys-His-Asp	amino acids and derivatives	C19H30N6O7S	1.37	2.70E-03	down
Ser-Glu-Tyr	amino acids and derivatives	C17H23N3O8	1.38	8.37E-05	up
His-Glu-Thr	amino acids and derivatives	C15H23N5O7	1.29	8.05E-03	up
Cys-Met-Ser	amino acids and derivatives	C11H21N3O5S2	1.59	1.06E-06	up
Lys-Lys	amino acids and derivatives	C12H26N4O3	1.46	4.53E-04	down
3-Nitrotyrosine	amino acids and derivatives	C9H10N2O5	1.59	2.20E-06	up
N-hexadecyl hydroxyproline	amino acids and derivatives	C21H41NO3	1.58	1.11E-06	down
N-Palmitoylglycine	amino acids and derivatives	C18H35NO3	1.57	4.15E-07	down
Thr-Met	amino acids and derivatives	C9H18N2O4S	1.60	2.30E-08	up
Tyr-His-Asp-Asp	amino acids and derivatives	C23H28N6O10	1.55	3.36E-06	up
TyrMe-Met-OH	amino acids and derivatives	C21H24N2O7S	1.57	6.13E-07	down
Ile-Glu-Val-Asp-Leu	amino acids and derivatives	C26H45N5O10	1.49	3.31E-06	down
Nap-Nap-OH	amino acids and derivatives	C31H24N2O6	1.55	1.95E-08	up
Asp-Glu-Ile-Asp	amino acids and derivatives	C19H30N4O11	1.55	6.02E-06	up
N-Formyl-DL-methionine	amino acids and derivatives	C6H11NO3S	1.45	1.07E-04	down
Asp-Gln-Ala-Asp	amino acids and derivatives	C16H25N5O10	1.18	9.34E-03	up
Arg-Gly-Arg	amino acids and derivatives	C14H29N9O4	1.57	3.87E-05	down
N'-Hydroxyneosaxitoxin	amino acids and derivatives	C10H17N7O6	1.54	4.19E-05	up
Gln-Thr-His	amino acids and derivatives	C15H24N6O6	1.46	1.67E-02	down
TyrMe-Asp-OH	amino acids and derivatives	C20H20N2O9	1.37	5.70E-04	up
TyrMe-TyrMe-OH	amino acids and derivatives	C26H26N2O8	1.51	4.14E-07	up
S-Inosyl-L-homocysteine	amino acids and derivatives	C14H19N5O6S	1.27	7.00E-03	down
Bis-gamma-glutamylcysteinylbis-beta-alanine	amino acids and derivatives	C22H36N6O12S2	1.48	9.48E-09	up
Thr-Trp-Met	amino acids and derivatives	C20H28N4O5S1	1.37	3.94E-03	up
Asn-Asp-Glu	amino acids and derivatives	C13H20N4O9	1.56	3.49E-06	down
Ser-Asp-Thr-Glu	amino acids and derivatives	C16H26N4O11	1.56	1.30E-08	up
Nap-Ser-OH	amino acids and derivatives	C21H18N2O7	1.60	1.02E-06	down
Glp-His-Pro-Gly	amino acids and derivatives	C18H24N6O6	1.29	1.93E-03	up
N-Acetylvanilalanine	amino acids and derivatives	C12H15NO5	1.59	1.42E-05	down
Gentianose	others	C18H32O16	1.37	7.71E-03	up
1-Kestose	others	C18H32O16	1.39	3.41E-03	up
Polydatin	others	C20H22O8	1.49	3.28E-05	up
Creatinol sulfate	others	C8H24N6O6S	1.24	1.14E-03	up
Eurycomalactone	others	C19H24O6	1.43	3.20E-03	up
Melezitose	others	C18H32O16	1.57	8.32E-05	up
Nicotinic acid	others	C6H5NO2	1.59	3.19E-06	down
Sucrose	others	C12H22O11	1.55	7.28E-05	up
Amylotetraose	others	C24H42O21	1.52	6.57E-06	up
Kojibiose	others	C12H22O11	1.38	1.89E-03	up
Erythritol	others	C4H10O4	1.54	2.39E-04	up
4-Methyl-5-thiazoleethanol	others	C6H9NOS	1.51	1.46E-05	down
Isopentyl gentiobioside	others	C17H32O11	1.21	3.09E-02	up
Heterodendrin	others	C11H19NO6	1.52	4.62E-07	down
4-Phenylsulfanyl-butan-2-one	others	C10H12OS	1.24	7.66E-04	up
Glucosamine	others	C6H13NO5	1.18	5.07E-02	up
Methoxyphedrine	others	C11H15NO2	1.58	1.39E-05	down
HoPhe-Ser-OH	others	C18H18N2O7	1.60	1.12E-11	up
2-O-alpha-L-Rhamnopyranosyl-D-glucopyranose	others	C12H22O10	1.57	5.38E-05	down
Cellobiose	others	C12H22O11	1.53	3.71E-04	up

Ethyl 4-O-(4-carboxy-3-hydroxy-3-methylbutanoyl)-beta-D-glucopyranoside	others	C14H24O10	1.50	5.81E-07	up
Phenothiazin-10-yl-piperidin-1-yl-methanone	others	C18H18N2OS	1.39	2.36E-04	down
2',4'-Dihydroxychalcone 4'-glucoside	others	C21H22O8	1.03	3.53E-02	up
alpha-1,5-L-Arabinotetraose	others	C20H34O17	1.47	6.80E-03	up
CID 74977408	others	C22H22O10	1.32	2.61E-02	up
[(2E)-2,11-dimethyl-7-methylidene-6,12-dioxo-5,14-dioxatricyclo[9.2.1.04,8]tetradeca-1(13),2-dien-9-yl] (E)-4-acetyloxy-2-methylbut-2-enoate	others	C22H24O8	1.13	2.10E-02	up
N,N',N''-triacetylchitotriose	others	C24H41N3O16	1.03	1.84E-02	down
Garcinone C	others	C23H26O7	1.49	1.67E-03	up
Ipecoside	others	C27H35NO12	1.34	4.62E-05	down
Acetaminophen glucuronide	others	C14H17NO8	1.60	3.85E-08	up
Raffinose	others	C18H32O16	1.58	6.97E-05	up
Trehalose	others	C12H22O11	1.55	9.15E-06	up
Stachyose	others	C24H42O21	1.53	2.60E-06	up
Maltopentaose	others	C30H52O26	1.40	5.38E-06	up
17-Hydroxypregnenolone sulfate	others	C21H32O6S	1.44	6.79E-03	up
2-(1,3-diphenyl-1H-pyrazol-4-yl)-3-(2-hydroxyethyl)-1,3-thiazolidin-4-one	others	C20H19N3O2S	1.49	1.24E-06	up
Trehalose-6-phosphate	others	C12H23O14P	1.58	6.22E-07	up
Sedoheptulose	others	C7H14O7	1.20	1.99E-02	up
3-[1-(3-Aminopropyl)-1H-indol-3-YL]-4-(1-methyl-1H-indol-3-YL)-1H-pyrrole-2,5-dione	others	C24H22N4O2	1.49	2.55E-03	up
Pentaerythritol tetrathioglycolate	others	C13H20O8S4	1.51	1.24E-04	up
Di-D-Fructofuranose 1,2':2,1'-Dianhydride	others	C12H20O10	1.18	2.68E-02	up
6'-O--D-Apiofuranosylsweroside	others	C21H30O13	1.47	6.37E-05	up
16alpha,17alpha-Dihydroxyprogesterone acetophenide	others	C29H36O4	1.13	2.20E-02	down
D-Fructose 6-Phosphate-Disodium Salt	others	C6H13O9P	1.57	1.27E-09	up
Melicopine	others	C17H15NO5	1.60	7.31E-09	up
Bryophyllin A	others	C26H32O8	1.45	7.73E-03	up
Farnesyl acetate	others	C17H28O2	1.56	2.01E-07	down
Rhapontin	others	C21H24O9	1.53	2.14E-08	down
Prim-O-glucosylcimifugin	others	C22H28O11	1.56	2.32E-06	down
N-cis-octadec-9Z-enoyl-L-Homoserine lactone	others	C22H39NO3	1.55	2.60E-08	down
D-Fructose	others	C6H12O6	1.60	3.74E-11	down
2-Oxonioacetate	others	C2H4O3	1.57	3.98E-05	down
5-hydroxy-2-(3-hydroxyphenyl)-7-methoxy-6-[3,4,5-trihydroxy-6-(hydroxymethyl)oxan-2-yl]-4H-chromen-4-one	others	C22H22O10	1.56	4.65E-07	up
Crenulatoside C	others	C29H48O11	1.57	9.62E-07	up
11a-Hydroxytetracycline	others	C22H24N2O9	1.53	2.34E-07	up
3,7-Dihydroxycoprostanic acid	others	C27H46O4	1.43	4.28E-03	up
1-Hydroxy-13-(4-oxoquinazolin-3-yl)-10-propan-2-yl-8,11-diazatetracyclo[6.6.1.02,7.011,15]pentadeca-2,4,6-triene-9,12-dione	others	C24H22N4O4	1.42	2.20E-03	up
Acetyl-maltose	others	C14H24O12	1.54	1.36E-08	down

(4-{4-[2-(gamma-L-glutamylamino)ethyl]phenoxymethyl}furan-2-yl)methanamine	others	C19H26N3O5+	1.13	6.87E-03	down
5-Acetoxy-7-[(6-O-alpha-L-rhamnopyranosyl-beta-D-glucopyranosyl)oxy]-4',6-dimethoxyflavone	others	C31H36O16	1.31	8.42E-05	up
5-hydroxy-2-(4-hydroxy-3-methoxyphenyl)-6,8-bis[3,4,5-trihydroxy-6-(hydroxymethyl)oxan-2-yl]-7-[(3,4,5-trihydroxy-6-methyloxan-2-yl)oxy]-4H-chromen-4-one	others	C34H42O20	1.58	4.29E-06	up
Dihydroferulic acid	organic acids	C10H12O4	1.58	9.06E-08	up
Succinylacetone	organic acids	C7H10O4	1.50	8.15E-06	up
S-Lactoylglutathione	organic acids	C13H21N3O8S	1.39	7.66E-04	up
2-Benzylsuccinic acid	organic acids	C11H12O4	1.07	8.60E-02	up
(S)-2-Amino-3-(1,3,5,7-pentahydro-2,4-dioxo-cyclopenta[E]pyrimidin-1-YL) proionic acid	organic acids	C10H13N3O4	1.11	1.46E-01	down
Ionomycin	organic acids	C41H72O9	1.14	2.55E-02	down
3-Hydroxydecanoic acid	organic acids	C10H20O3	1.18	4.95E-03	down
Isobutyric acid	organic acids	C4H8O2	1.15	1.37E-02	down
Methyl 2,5-dihydroxycinnamate	organic acids	C10H10O4	1.56	5.17E-10	up
Dihomo-g-Linolenic Acid-d6	organic acids	C20H28D6O2	1.31	7.98E-03	down
3,6,11,12-Tetrakis(acetyloxy)-5-(4-hydroxyphenyl)-8-oxatricyclo[7.4.0.0^{2,7}]trideca-1(13),2,4,6,9,11-hexaen-4-yl acetate	organic acids	C28H22O12	1.27	3.14E-03	up
TRIETHYL PHOSPHATE	organic acids	C6H15O4P	1.57	1.48E-05	up
3,4,5-trihydroxy-6-[4-(1-hydroxy-3-oxobutyl)-2-methoxyphenoxy]oxane-2-carboxylic acid	organic acids	C17H22O10	1.55	4.57E-08	up
Flavone base + 3O, O-HexA, C-Hex, C-Hex	organic acids	C33H38O21	1.40	5.08E-03	up
6-({2-[4-ethyl-3-(sulfooxy)phenyl]-3,5-dihydroxy-3,4-dihydro-2H-1-benzopyran-7-yl}oxy)-3,4,5-trihydroxyoxane-2-carboxylic acid	organic acids	C23H26O14S	1.05	7.43E-02	up
3,8-Dihydroxy-1-pentanoyl-6-pentyl-11H-dibenzo(b,e)(1,4)dioxepin-11-one	organic acids	C23H26O6	1.54	1.85E-03	up
Lobaric acid	organic acids	C25H28O8	1.50	4.67E-05	up
Ciprostene	organic acids	C22H36O4	1.53	4.91E-04	down
N-Tris(hydroxymethyl)methyl-2-aminoethanesulfonic acid	organic acids	C6H15NO6S	1.51	1.27E-03	down
2-methyl citric acid	organic acids	C7H10O7	1.51	4.13E-07	down
(2E)-3-[4-({2-O-[(2S,3R,4R)-3,4-Dihydroxy-4-(hydroxymethyl)tetrahydro-2-furanyl]-beta-D-glucopyranosyl}oxy)-3-methoxyphenyl]acrylic acid	organic acids	C21H28O13	1.59	2.79E-07	up
6-(2,4-dihydroxyphenyl)-2-{4-[(E)-2-(2,4-dihydroxyphenyl)ethenyl]-2,6-dihydroxyphenyl}-4-methylcyclohex-3-ene-1-carboxylic acid	organic acids	C28H26O8	1.57	1.79E-05	up
{[12-(6,11-dihydroxy-2,2,5-trimethyl-10-oxo-3,4,5,10-tetrahydro-2H-1-oxa-5-azatetraphen-4-yl)-6,11-dihydroxy-2,5-dimethyl-10-oxo-5,10-dihydro-2H-1-oxa-5-azatetraphen-2-yl]methoxy}sulfonic acid	organic acids	C38H34N2O12S	1.45	6.39E-04	up

6-(2,4-dihydroxyphenyl)-2-{3-[(2E)-3-(2,4-dihydroxyphenyl)prop-2-enoyl]-2,6-dihydroxyphenyl}-4-methylcyclohex-3-ene-1-carboxylic acid	organic acids	C29H26O9	1.59	7.18E-08	up
{5-[2,4-dihydroxy-3-(3-methylbut-2-en-1-yl)benzoyl]-6-(2,4-dihydroxyphenyl)-4-{4-[(E)-2-(2,4-dihydroxyphenyl)ethenyl]-2,6-dihydroxyphenyl}-2-methylcyclohex-2-en-1-yl}oxidanesulfonic acid	organic acids	C39H38O13S	1.50	5.75E-07	up
9-Oxo-nonanoic acid	organic acids	C9H16O3	1.52	2.31E-05	down
2-(6-Carboxy-3,4,5-trihydroxyoxan-2-yl)propanedioic acid	organic acids	C9H12O10	1.43	8.65E-06	down
{[(2E)-4-{3-[6-(2,4-dihydroxyphenyl)-2-{4-[(E)-2-(2,4-dihydroxyphenyl)ethenyl]-2,6-dihydroxyphenyl}-4-methylcyclohex-3-ene-1-carbonyl]-2,6-dihydroxyphenyl}-2-methylbut-2-en-1-yl]oxy}sulfonic acid	organic acids	C39H38O13S	1.54	2.19E-07	up
{4-[2,3-dioxo-3-(2,4,6-trihydroxy-3-methoxyphenyl)propyl]-2-methoxyphenyl}oxidanesulfonic acid	organic acids	C17H16O11S	1.50	2.53E-07	up
[4-(5-Hydroxy-7-methoxy-8-methyl-4-oxochromen-3-yl)-2-methoxyphenyl] hydrogen sulate	organic acids	C18H16O9S	1.59	4.64E-06	down
2-amino-4-({1-[(carboxymethyl)-C-hydroxycarbonimidoyl]-2-{[2-hydroxy-1-(4-methoxyphenyl)-3-oxopropyl]sulfanyl}ethyl}-C-hydroxycarbonimidoyl)butanoic acid	organic acids	C20H27N3O9S	1.35	2.95E-04	up
({1-[2,4-dihydroxy-5-(3-methylbut-2-en-1-yl)phenyl]-3-[3,4-dihydroxy-5-(3-methylbut-2-en-1-yl)phenyl]-1-oxopropan-2-yl}oxy)sulfonic acid	organic acids	C25H30O9S	1.34	5.54E-04	up
[2-hydroxy-6-(3-methylbut-2-en-1-yl)-4-{1-oxo-1-[2,4,6-trihydroxy-3-(3-methylbut-2-en-1-yl)phenyl]propan-2-yl}phenyl]oxidanesulfonic acid	organic acids	C25H30O9S	1.60	2.81E-06	up
3,4,5-trihydroxy-6-[4-(6-hydroxy-4-{5-[(E)-2-[6-hydroxy-3-(3-hydroxyphenyl)-2-phenyl-2,3-dihydro-1-benzofuran-4-yl]ethenyl]-2-(4-hydroxyphenyl)-1-benzofuran-3-yl}-3-(3-hydroxyphenyl)-2,3-dihydro-1-benzofuran-2-yl)phenoxy]oxane-2-carboxylic acid	organic acids	C62H48O15	1.36	2.03E-03	up
6-({4-[2-(3,4-dihydroxyphenyl)-3,5,7-trihydroxy-3,4-dihydro-2H-1-benzopyran-8-yl]-3,7-dihydroxy-2-(3-hydroxyphenyl)-8-[3,4,5-trihydroxy-6-(hydroxymethyl)oxan-2-yl]-3,4-dihydro-2H-1-benzopyran-5-yl}oxy)-3,4,5-trihydroxyoxane-2-carboxylic acid	organic acids	C42H44O22	1.45	1.75E-06	down
6-({8-[5,7-dihydroxy-2-(4-hydroxyphenyl)-4-oxo-3,4-dihydro-2H-1-benzopyran-3-yl]-2-(3,4-dihydroxyphenyl)-7-hydroxy-4-oxo-3,4-dihydro-2H-1-benzopyran-5-yl}oxy)-3,4,5-trihydroxyoxane-2-carboxylic acid	organic acids	C36H30O17	1.37	3.76E-03	up
2-amino-4-({1-[(carboxymethyl)-C-hydroxycarbonimidoyl]-2-{[1-hydroxy-1-(4-hydroxy-3-	organic acids	C21H29N3O10S	1.41	2.63E-03	down

methoxyphenyl)-3-oxobutan-2-yl]sulfanyl}ethyl}-C-hydroxycarbonimidoyl)butanoic acid					
[(2-{2-[2-(2,4-dihydroxyphenyl)-5,7-dihydroxy-6-(3-methylbut-2-en-1-yl)-4-oxo-4H-chromen-3-yl]ethylidene}-6-methylhept-5-en-1-yl)oxy]sulfonic acid	organic acids	C30H34O10S	1.41	1.07E-03	down
2-amino-4-({1-[(carboxymethyl)-C-hydroxycarbonimidoyl]-2-{[1-(3,5-dihydroxyphenyl)-2-hydroxy-2-(3-hydroxy-4-methoxyphenyl)ethyl]sulfanyl}ethyl}-C-hydroxycarbonimidoyl)butanoic acid	organic acids	C25H31N3O11S	1.59	3.61E-06	down
6-{4-[3-(3,5-dihydroxyphenyl)-6-hydroxy-4-{5-[(E)-2-[6-hydroxy-3-(3-hydroxyphenyl)-2-phenyl-2,3-dihydro-1-benzofuran-4-yl]ethenyl]-2-(4-hydroxyphenyl)-1-benzofuran-3-yl}-2,3-dihydro-1-benzofuran-2-yl]phenoxy}-3,4,5-trihydroxyoxane-2-carboxylic acid	organic acids	C62H48O16	1.28	1.04E-02	down
6-({6-[(acetyloxy)methyl]-2,4,5-trihydroxyoxan-3-yl}oxy)-3,4,5-trihydroxyoxane-2-carboxylic acid	organic acids	C14H22O13	1.55	6.04E-08	down
6-[6-(2,4-dihydroxyphenyl)-2-{4-[(E)-2-(2,4-dihydroxyphenyl)ethenyl]-2,6-dihydroxyphenyl}-4-methylcyclohex-3-ene-1-carbonyloxy]-3,4,5-trihydroxyoxane-2-carboxylic acid	organic acids	C34H34O14	1.46	5.14E-05	down
3,4,5-trihydroxy-6-{[5-hydroxy-4-oxo-2-phenyl-8-(3,4,5-trihydroxyoxan-2-yl)-4H-chromen-7-yl]oxy}oxane-2-carboxylic acid	organic acids	C26H26O14	1.52	1.18E-04	down
6-{6-[6-(2,4-dihydroxyphenyl)-2-{4-[(E)-2-(2,4-dihydroxyphenyl)ethenyl]-2,6-dihydroxyphenyl}-6-hydroxy-4-methylcyclohex-3-ene-1-carbonyl]-3-hydroxy-2-(3-methylbut-2-en-1-yl)phenoxy}-3,4,5-trihydroxyoxane-2-carboxylic acid	organic acids	C45H46O16	1.47	6.08E-03	up
6-{2-[2-(2,4-dihydroxyphenyl)-2-oxoethyl]-4-[(3,3-dimethyloxiran-2-yl)methyl]-5-hydroxy-3-methoxyphenoxy}-3,4,5-trihydroxyoxane-2-carboxylic acid	organic acids	C26H30O13	1.42	1.28E-07	up
2-amino-4-({1-[(carboxymethyl)-C-hydroxycarbonimidoyl]-2-[(4-{3,5-dihydroxy-2,2-dimethyl-8-oxo-6-propyl-2H,3H,4H,8H-pyrano[3,2-g]chromen-10-yl}-4-oxobutyl)sulfanyl]ethyl}-C-hydroxycarbonimidoyl)butanoic acid	organic acids	C31H41N3O12S	1.41	6.29E-04	up
6-[(2-{4-[6-carboxy-5-(2,4-dihydroxyphenyl)-3-methylcyclohex-2-en-1-yl]-3,5-dihydroxyphenyl}-7-(3-methylbut-2-en-1-yl)-1-benzofuran-6-yl)oxy]-3,4,5-trihydroxyoxane-2-carboxylic acid	organic acids	C39H40O14	1.45	3.13E-04	up
2-amino-4-({1-[(carboxymethyl)-C-hydroxycarbonimidoyl]-2-{[1,3-dihydroxy-4-(7-methoxy-2-oxo-2H-chromen-8-yl)-2-methylbutan-2-yl]sulfanyl}ethyl}-C-hydroxycarbonimidoyl)butanoic acid	organic acids	C25H33N3O11S	1.52	2.48E-04	down
6-[4-(3-{3-[6-carboxy-5-(2,4-dihydroxyphenyl)-3-methylcyclohex-2-en-1-yl]-2,4-dihydroxyphenyl}-3-	organic acids	C35H36O15	1.40	7.84E-04	up

oxopropyl)-3-hydroxyphenoxy]-3,4,5-trihydroxyoxane-2-carboxylic acid					
(S,S)-(-)-Hydrobenzoin	benzene and substituted derivatives	C14H14O2	1.50	6.36E-03	down
Red Violet 2RN Acid Anthraquinone	benzene and substituted derivatives	C21H13NO4	1.55	3.25E-06	up
4-phenyl-5-methyl-1,2,3-Thiadiazole	benzene and substituted derivatives	C9H8N2S	1.39	9.75E-06	up
Diphenylmethylene-Glycine benzyl ester	benzene and substituted derivatives	C22H19NO2	1.35	1.78E-06	down
1-Benzyl-4-hydroxy-5-oxo-2,5-dihydro-1H-pyrrole-3-carboxylic acid ethyl ester	benzene and substituted derivatives	C14H15NO4	1.56	7.71E-05	up
Glucovanillin	benzene and substituted derivatives	C14H18O8	1.21	3.51E-08	up
Isofenphos-methyl	benzene and substituted derivatives	C14H22NO4PS	1.54	4.61E-06	down
Populin	benzene and substituted derivatives	C20H22O8	1.12	1.38E-04	up
9,10-Anthracenediol, 9,10-dihydro-9,10-diphenyl-	benzene and substituted derivatives	C26H20O2	1.21	1.22E-03	up
2-(1,4-Diazepan-1-yl)benzo[d]thiazole	benzene and substituted derivatives	C12H15N3S	1.48	5.57E-03	up
5-(2,4-dimethoxybenzylidene)pyrimidine-2,4,6(1H,3H,5H)-trione	benzene and substituted derivatives	C13H12N2O5	1.12	2.15E-03	up
2-NP-Amoz	benzene and substituted derivatives	C15H18N4O5	1.54	2.08E-08	up
Gomphrenin II	benzene and substituted derivatives	C33H32N2O15	1.13	2.84E-02	up
2-Amino-5-methyl-4-phenylthiazole	benzene and substituted derivatives	C10H10N2S	1.11	8.50E-03	up
3-[(2S,3R,4S,5S,6R)-4,5-dihydroxy-3-[(2R,3R,4R,5R,6S)-3,4,5-trihydroxy-6-methyloxan-2-yl]oxy-6-[[(2R,3R,4R,5R,6S)-3,4,5-trihydroxy-6-methyloxan-2-yl]oxymethyl]oxan-2-yl]oxy-5,7-dihydroxy-2-(4-hydroxy-3-methoxyphenyl)-6-methoxychromen-4-one	benzene and substituted derivatives	C35H44O21	1.40	8.95E-06	down
cyanidin 3-O-glucoside-7-O-(6-O-(4-O-(glucosyl)-oxybenzoyl)-glucoside)	benzene and substituted derivatives	C40H44O23	1.02	3.04E-02	up
6,8-bis[3,7-dihydroxy-2-(3-hydroxyphenyl)-3,4-dihydro-2H-1-benzopyran-4-yl]-2-(4-hydroxy-3-methoxyphenyl)-3,4-dihydro-2H-1-benzopyran-3,5,7-triol	benzene and substituted derivatives	C46H40O14	1.13	1.89E-02	down
3,4,5-trihydroxy-6-[4-(4-methyl-3-oxopent-1-en-1-yl)phenoxy]oxane-2-carboxylic acid	benzene and substituted derivatives	C18H22O8	1.34	3.16E-04	up
Syringic acid sulfate	benzene and substituted derivatives	C9H10O8S	1.54	6.78E-05	down
Cephalomannine	benzene and substituted derivatives	C45H53NO14	1.37	6.86E-03	down
Pyribenzoxim	benzene and substituted derivatives	C32H27N5O8	1.58	6.16E-06	up

Sambacolignoside	benzene and substituted derivatives	C43H54O22	1.58	3.03E-11	up
Orthosulfamuron	benzene and substituted derivatives	C16H20N6O6S	1.48	2.18E-03	up
Trichotomine	benzene and substituted derivatives	C30H20N4O6	1.52	3.05E-05	up
Acrivastine	benzene and substituted derivatives	C22H24N2O2	1.55	2.81E-06	down
Sulfinpyrazone	benzene and substituted derivatives	C23H20N2O3S	1.48	1.47E-07	down
2-[(1R,5S,6R,11R,13R,16S)-6-(furan-3-yl)-11-hydroxy-1,5,15,15-tetramethyl-8,14,17-trioxo-7-oxatetracyclo[11.3.1.02,11.05,10]heptadec-9-en-16-yl]acetic acid	benzene and substituted derivatives	C26H30O8	1.55	1.04E-07	up
Mollicellin I	benzene and substituted derivatives	C21H22O6	1.41	5.34E-03	up
6-[3,7-dihydroxy-2-(3-hydroxyphenyl)-3,4-dihydro-2H-1-benzopyran-4-yl]-8-[3,7-dihydroxy-2-(4-hydroxy-3-methoxyphenyl)-3,4-dihydro-2H-1-benzopyran-4-yl]-2-(3-hydroxyphenyl)-3,4-dihydro-2H-1-benzopyran-3,5,7-triol	benzene and substituted derivatives	C46H40O14	1.50	1.02E-05	down
6-Caffeoylsucrose	benzene and substituted derivatives	C21H28O14	1.39	5.63E-04	up
8-[3,7-dihydroxy-2-(4-hydroxy-3-methoxyphenyl)-3,4-dihydro-2H-1-benzopyran-4-yl]-2-(3,4-dihydroxyphenyl)-6-[2-(3,4-dihydroxyphenyl)-3,7-dihydroxy-3,4-dihydro-2H-1-benzopyran-4-yl]-3,4-dihydro-2H-1-benzopyran-3,5,7-triol	benzene and substituted derivatives	C46H40O16	1.47	1.84E-05	up
5-hydroxy-2-(4-hydroxy-3,5-dimethoxyphenyl)-7-methoxy-6-(3-methylbut-2-en-1-yl)-3,4-dihydro-2H-1-benzopyran-4-one	benzene and substituted derivatives	C23H26O7	1.46	9.02E-04	up
4-[2-(3,4-dihydroxyphenyl)-3,5,7-trihydroxy-3,4-dihydro-2H-1-benzopyran-8-yl]-2-(4-hydroxy-3-methoxyphenyl)-8-[3,4,5-trihydroxy-6-(hydroxymethyl)oxan-2-yl]-3,4-dihydro-2H-1-benzopyran-3,5,7-triol	benzene and substituted derivatives	C37H38O17	1.49	2.22E-06	up
NCGC00380161-01_C20H28O12_6-O-(Phenylacetyl)-alpha-D-glucopyranosyl alpha-D-glucopyranoside	benzene and substituted derivatives	C20H28O12	1.48	1.76E-04	up
5,7-dihydroxy-2-phenyl-6,8-bis[3,4,5-trihydroxy-6-(hydroxymethyl)oxan-2-yl]-3,4-dihydro-2H-1-benzopyran-4-one	benzene and substituted derivatives	C27H32O14	1.57	5.44E-05	up
Glabranin	flavonoids	C20H20O4	1.57	9.45E-07	down
Diosmin	flavonoids	C28H32O15	1.28	2.96E-05	up
4H-1-Benzopyran-4-one, 5-hydroxy-8-(5-(5-hydroxy-7-methoxy-4-oxo-4H-1-benzopyran-2-yl)-2-methoxyphenyl)-7-methoxy-2-(4-methoxyphenyl)-, (+)-	flavonoids	C34H26O10	1.44	2.48E-03	up
Vitexin 2''-O-rhamnoside	flavonoids	C27H30O14	1.58	3.46E-07	up
Tricin-7-O-(2''-Sinapoyl)glucuronide	flavonoids	C34H32O17	1.26	2.75E-03	up
4',5-Dihydroxy-3',5'-dimethoxyflavone	flavonoids	C17H14O6	1.57	3.08E-03	down

3-O-b-D-Galactopyranosylproanthocyanidin A5'	flavonoids	C36H34O17	1.05	8.61E-06	up
Peonidin 3-(6"-acetylglucoside)	flavonoids	C24H25O12+	1.58	8.65E-08	up
Flavone base + 3O, O-HexA-FeruloylHexA	flavonoids	C37H34O20	1.52	2.64E-04	up
3"-O-L-Rhamnopyranosylastragalin	flavonoids	C27H30O15	1.48	5.42E-09	up
Isoginkgetin	flavonoids	C32H22O10	1.55	3.18E-06	up
Spinosin	flavonoids	C28H32O15	1.18	1.74E-03	up
3,6-Diglucopyranosyl-5,7-dihydroxy-4'-methoxyflavone	flavonoids	C28H32O15	1.53	3.99E-06	up
ent-Fisetinidol-(4beta->8)-catechin-(6->4beta)-ent-fisetinidol	flavonoids	C45H38O16	1.31	3.20E-05	up
3,7-Di-O-methylquercetin	flavonoids	C17H14O7	1.57	3.39E-06	down
Rutin	flavonoids	C27H30O16	1.49	3.61E-04	up
5,7-Dimethoxyflavone	flavonoids	C17H14O4	1.54	1.79E-04	down
Naringin 4'-glucoside	flavonoids	C33H42O19	1.48	4.56E-06	up
Dactilin	flavonoids	C28H32O17	1.07	1.14E-02	up
Kaempferol3-rhamninoside	flavonoids	C33H40O19	1.42	1.97E-05	down
Quercetin 3-xylosyl-(1->6)-glucoside	flavonoids	C26H28O16	1.43	1.62E-05	up
7-hydroxy-2-(4-hydroxy-3-methoxyphenyl)-3,5-bis({[3,4,5-trihydroxy-6-(hydroxymethyl)oxan-2-yl]oxy})-1lambdachromen-1-ylium	flavonoids	C28H33O16	1.46	8.09E-06	up
Betaine	alkaloids	C5H11NO2	1.58	1.97E-05	down
Lumichrome	alkaloids	C12H10N4O2	1.54	3.78E-04	down
Dopamine	alkaloids	C8H11NO2	1.39	2.53E-04	up
(2S,4R)-4-(9H-Pyrido[3,4-b]indol-1-yl)-1,2,4-butanetriol	alkaloids	C15H16N2O3	1.33	8.58E-03	down
Salicylamide	alkaloids	C7H7NO2	1.50	3.14E-06	down
Trans-Zeatin	alkaloids	C10H13N5O	1.57	2.95E-07	down
N-Feruloyl dopamine, trans-	alkaloids	C18H19NO5	1.58	9.33E-06	up
Arecaidine	alkaloids	C7H11NO2	1.15	6.47E-04	up
Indole-5-carboxylic acid	alkaloids	C9H7NO2	1.05	5.37E-04	down
DIMBOA glucoside	alkaloids	C15H19NO10	1.32	4.82E-03	up
1-Naphthylamine	alkaloids	C10H9N	1.44	4.19E-06	down
Tifentai	alkaloids	C27H28N2O4	1.46	8.83E-03	down
methyl (1R,12R,14S,15E)-12-[(1R,15S,17S,18S)-17-ethyl-3,13-diazapentacyclo[13.3.1.02,10.04,9.013,18]nonadeca-2(10),4(9),5,7-tetraen-6-yl]-15-ethylidene-17-methyl-10,17-diazatetracyclo[12.3.1.03,11.04,9]octadeca-3(11),4,6,8-tetraene-18-carboxylate	alkaloids	C40H48N4O2	1.51	5.91E-04	down
Geissospermine	alkaloids	C40H48N4O3	1.47	3.19E-03	down
(e)-2-[(2s,3s)-3-Ethyl-8-methoxy-1,2,3,4,6,7,12,12b-octahydroindolo[3,2-h]quinolizin-2-yl]-3-methoxyprop-2-enoic acid methyl ester	alkaloids	C23H30N2O4	1.43	1.04E-02	down
[(2R,3S,4S,5R,6R)-6-[(2S,3S,4S,5R)-3,4-dihydroxy-2,5-bis(hydroxymethyl)oxolan-2-yl]oxy-3,4,5-trihydroxyoxan-2-yl]methyl (E)-3-(4-hydroxy-3,5-dimethoxyphenyl)prop-2-enoate	phenolic acids	C23H32O15	1.43	9.71E-03	up
Usnic acid	phenolic acids	C18H16O7	1.43	7.72E-03	down
Gastrodin	phenolic acids	C13H18O7	1.55	8.93E-06	down
CID 487435	phenolic acids	C17H20O9	1.53	1.09E-05	up
Dimethyl fumarate	phenolic acids	C6H8O4	1.53	6.92E-05	up

3,4,5-Trimethoxycinnamic acid	phenolic acids	C12H14O5	1.12	2.15E-06	down
1,7-Bis(4-hydroxyphenyl)-3-heptanone	phenolic acids	C19H22O3	1.54	3.46E-06	down
4-Ethylbenzoic acid	phenolic acids	C9H10O2	1.30	2.31E-03	down
1-Caffeoyl-beta-D-glucose	phenolic acids	C15H18O9	1.19	8.95E-04	up
Trihydroxycinnamoylquinic acid	phenolic acids	C16H20O10	1.36	2.18E-05	up
cis-coumarinic acid-beta-D-glucoside	phenolic acids	C15H18O8	1.44	1.71E-04	up
4-Hydroxybenzoic acid	phenolic acids	C7H6O3	1.59	5.44E-06	down
Rosmarinic acid	phenolic acids	C18H16O8	1.60	5.88E-07	up
Forsythiaside	phenolic acids	C29H36O15	1.56	2.40E-06	down
1-(1Z-hexadecenyl)-sn-glycero-3-phosphocholine	GP	C24H50NO6P	1.50	6.49E-07	down
1-Stearoyl-2-oleoyl-sn-glycero-3-phosphocholine	GP	C44H86NO8P	1.38	7.17E-03	up
2,3-dichloro-N-[3-(5-methyl-1,3-benzoxazol-2-yl)phenyl]benzamide	GP	C21H14Cl2N2O2	1.56	3.15E-07	up
1-(9Z,12Z-octadecadienoyl)-glycero-3-phosphate	GP	C21H39O7P	1.56	2.06E-06	up
1,2-Dimyristoyl-sn-glycero-3-phosphocholine	GP	C36H72NO8P	1.56	8.12E-05	down
PA(14:0/22:4(7Z,10Z,13Z,16Z))	GP	C39H69O8P	1.23	1.92E-03	down
PE-NMe(15:0/18:3(6Z,9Z,12Z))	GP	C39H72NO8P	1.28	1.17E-04	down
PA(18:3(6Z,9Z,12Z)/24:1(15Z))	GP	C45H81O8P	1.50	1.43E-04	down
LPE(0:0/22:5)	GP	C27H46NO7P	1.45	2.44E-03	down
PC(18:2(9Z,12Z)/22:2(13Z,16Z))	GP	C48H88NO8P	1.58	1.34E-05	down
1-(9Z,12Z-octadecadienoyl)-2-tetradecanoyl-glycero-3-phosphocholine	GP	C40H76NO8P	1.43	4.51E-04	down
(-)-Salsoline	heterocyclic compounds	C11H15NO2	1.46	1.01E-02	down
Oxypurinol	heterocyclic compounds	C5H4N4O2	1.56	2.10E-05	down
5,5-Dimethyl-2-(piperazin-1-yl)-4,5-dihydrothiazole	heterocyclic compounds	C9H17N3S	1.48	4.39E-05	down
(5R,6E)-3-[(E)-2-acetamidoethenyl]sulfinyl-6-(1-hydroxypropan-2-ylidene)-7-oxo-1-azabicyclo[3.2.0]hept-2-ene-2-carboxylic acid	heterocyclic compounds	C14H16N2O6S	1.39	1.80E-05	up
Citbismine C	heterocyclic compounds	C37H36N2O11	1.58	1.86E-07	up
2,8-Quinolinediol	heterocyclic compounds	C9H7NO2	1.57	4.46E-06	down
Canaliculatol	heterocyclic compounds	C42H32O9	1.56	7.85E-07	up
1-Isomangostin	heterocyclic compounds	C24H26O6	1.44	3.92E-04	up
Acteoside	heterocyclic compounds	C29H36O15	1.56	1.71E-05	up
Leucovorin	heterocyclic compounds	C20H23N7O7	1.58	5.05E-05	up
Citbismine F	heterocyclic compounds	C36H34N2O10	1.34	3.76E-05	up
N2,N2-Dimethylguanosine	nucleotides and derivatives	C12H17N5O5	1.45	4.67E-06	down
2'-O-Methyladenosine	nucleotides and derivatives	C11H15N5O4	1.04	9.31E-02	down
3'-Deoxyguanosine	nucleotides and derivatives	C10H13N5O4	1.33	5.42E-03	down
Thymidine	nucleotides and derivatives	C10H14N2O5	1.42	6.43E-05	up
Lamivudine	nucleotides and derivatives	C8H11N3O3S	1.51	1.91E-05	up
dTDP-3-N,N-dimethylamino-4-oxo-2,3,6-trideoxy-alpha-D-glucose	nucleotides and derivatives	C18H28N3O13P2-	1.32	4.92E-05	up
dTDP-4-(methylamino)-2,3,4,6-tetradeoxy-alpha-D-glucose	nucleotides and derivatives	C17H29N3O12P2	1.55	1.12E-06	up
Adenosine-5'-phosphosulfate	nucleotides and derivatives	C10H14N5O10PS	1.59	2.60E-07	up
UDP-2-acetamido-2,6-dideoxy-alpha-D-xylo-hex-4-ulose(2-)	nucleotides and derivatives	C17H23N3O16P2-2	1.50	3.01E-03	up
GDP-4-oxo-L-rhamnose	nucleotides and derivatives	C16H23N5O15P2	1.60	2.70E-06	up
GDP-D-glycero-alpha-D-manno-heptose	nucleotides and derivatives	C17H27N5O17P2	1.43	1.03E-08	up

2,4-Quinolinediol	alcohol and amines	C9H7NO2	1.49	3.75E-06	down
Triethanolamine	alcohol and amines	C6H15NO3	1.59	2.29E-07	down
Aminopentol	alcohol and amines	C22H47NO5	1.49	2.63E-07	down
N-(tert-butyl)-2-(2-thienyl)acetamide	alcohol and amines	C10H15NOS	1.23	2.46E-02	down
3'-O-beta-Glucopyranosyl-stilbericoside	alcohol and amines	C20H30O15	1.40	1.53E-03	up
Glc4Me(a1-4)Glc(a)-O-Me	alcohol and amines	C14H26O11	1.35	1.03E-02	up
Guaiacol beta-primeveroside	alcohol and amines	C18H26O11	1.50	3.15E-06	up
beta-D-apiofuranosyl-(1->6)-D-glucose	alcohol and amines	C11H20O10	1.20	2.45E-03	down
6-Methylcoumarin	lignans and Coumarins	C10H8O2	1.19	4.10E-03	up
Matairesinol	lignans and Coumarins	C20H22O6	1.53	1.50E-07	up
Osthenol	lignans and Coumarins	C14H14O3	1.13	9.74E-04	up
Simplexoside	lignans and Coumarins	C26H30O11	1.59	3.29E-05	up
Syringaresinol	lignans and Coumarins	C22H26O8	1.47	9.16E-07	down
Podorhizol beta-D-glucoside	lignans and Coumarins	C28H34O13	1.52	6.31E-04	up
(-)-Hydroxymatairesinol	lignans and Coumarins	C20H22O7	1.29	1.05E-02	down
(+)-sesaminol 2-O-beta-D-gentiobioside	lignans and Coumarins	C32H38O17	1.46	3.22E-03	up
Corosolic acid	terpenoids	C30H48O4	1.28	7.01E-03	down
Harpagoside	terpenoids	C24H30O11	1.45	2.41E-05	up
Safrole	terpenoids	C10H10O2	1.59	3.21E-11	down
Paeoniflorin	terpenoids	C23H28O11	1.57	4.51E-05	up
(2S,9R,13R,17S)-4,15-dimethoxy-2,6,14,17-tetramethyl-10-oxatetracyclo[7.7.1.02,7.013,17]heptadeca-4,14-diene-3,11,16-trione	terpenoids	C22H28O6	1.46	7.31E-03	up
Ingenol	terpenoids	C20H28O5	1.39	1.97E-02	down
(-)-Oleoside 11-methyl ester	terpenoids	C17H24O11	1.53	8.89E-07	up
orthosiphol D	terpenoids	C31H36O9	1.49	8.63E-05	up
9,12-Octadecadiynoic Acid	lipids	C18H28O2	1.19	9.51E-03	down
Palmitoyl Serinol	lipids	C19H39NO3	1.48	1.77E-04	down
Choline Alfoscerate	lipids	C8H20NO6P	1.59	1.33E-05	up
17-(4-Hydroxyphenyl)heptadecanoic acid	lipids	C23H38O3	1.17	1.84E-05	down
12-HOME	lipids	C18H34O3	1.46	2.16E-05	down
(±)9-HpODE	lipids	C18H32O4	1.11	1.48E-02	down
2-Hydroxyoctadecanoic acid	lipids	C18H36O3	1.54	2.72E-06	down
Tributyrin	GL	C15H26O6	1.55	5.26E-09	up
2-Arachidonoyl glycerol-d5	GL	C23H38O4	1.16	1.67E-03	up
TG(16:1(9Z)/14:0/18:1(9Z))	GL	C51H94O6	1.02	6.14E-02	down
Glycerol trisinapate	GL	C36H38O15	1.57	2.11E-09	up
Carnitine C4:DC	FA	C11H19NO6	1.46	3.71E-07	down
5-Octadecanoyloxy-octadecanoic acid	FA	C36H70O4	1.54	3.06E-05	down
N-(dodecanoyl)-sphing-4-enine-1-phosphocholine	SL	C35H71N2O6P	1.25	7.11E-03	up
Procyanidin B1	tannins	C30H26O12	1.53	1.34E-03	up
